# Non-painful, Non-itchy Monkeypox Virus Rash: A Case Report

**DOI:** 10.7759/cureus.32342

**Published:** 2022-12-09

**Authors:** Sethi Ashish, Robert Patterson, Alyssa K Tomsey

**Affiliations:** 1 Infectious Disease, University of Missouri-Kansas City (UMKC) School of Medicine, Kansas City, USA; 2 Infectious Disease, University Health Truman Medical Center/Saint Luke's Hospital, Kansas City, USA; 3 Pathology, Butler Memorial Hospital, Butler, USA; 4 Emergency Medicine, Butler Health System, Butler Memorial Hospital, Butler, USA

**Keywords:** monkeypox virus, monkeypox virus rash, acam2000, jynneos, men who have sex with other men (msm), nigeria, hiv, tecovirimat, small pox

## Abstract

Monkeypox virus (MPVX) is a double-stranded deoxyribonucleic acid (DNA) virus from the genus *Orthopoxvirus*, family Poxviridae. Many cases of MPVX infection are currently seen worldwide. Although the majority of cases reported are not severe, basic precautionary measures are key to preventing this infection from turning into a catastrophic pandemic. We report a case of a 54-year-old male with no high-risk behavior who developed a sudden uncharacteristic monkeypox virus rash while on a flight from Nigeria.

## Introduction

Monkeypox virus (MPVX), a rare zoonotic infection, was first reported in 1958 as an outbreak of a pox-like disease in monkeys in Copenhagen, Denmark [1]. Nearly after a decade, on September 1, 1970, the first case of human MPXV infection was diagnosed in an infant who was admitted to the hospital in the Democratic Republic of Congo (DRC). The boy presented with a smallpox-like disease from which MPXV was isolated [2,3]. MPXV is caused by a double-stranded deoxyribonucleic acid (DNA) virus from the same family of Orthomyxovirus that also causes smallpox. Currently, the World Health Organization (WHO) has recorded approximately 70,000 diagnosed MPXV infections and 26 mortalities worldwide. There are two forms of MPXV: Clade 1 and Clade 2. We report a case of serologically diagnosed MPXV infection with an unusual dermatological clinical presentation.

## Case presentation

**Figure 1:**
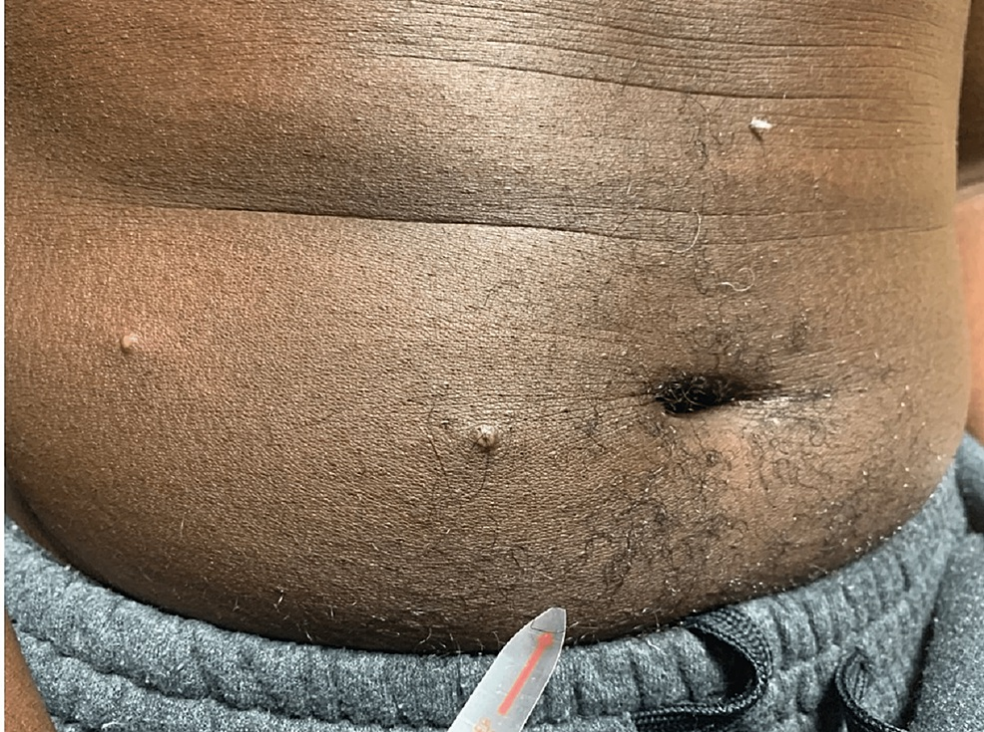
Non-itchy and non-painful MPVX rash MPVX: monkeypox virus

**Figure 2:**
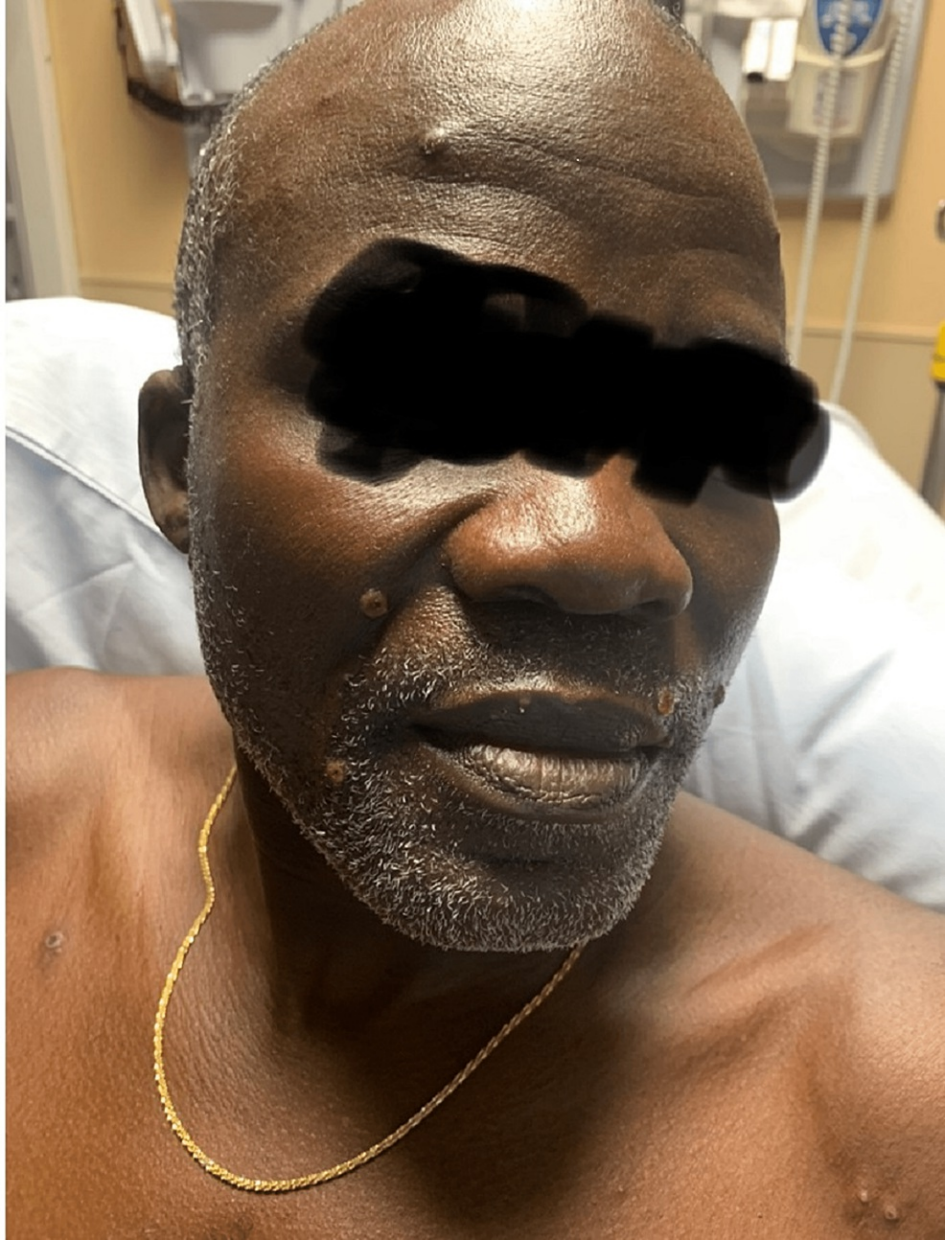
MPVX rash: umbilicated pustule, 3-4 mm in diameter (facial lesion) MPVX: monkeypox virus, mm: millimeter

**Figure 3:**
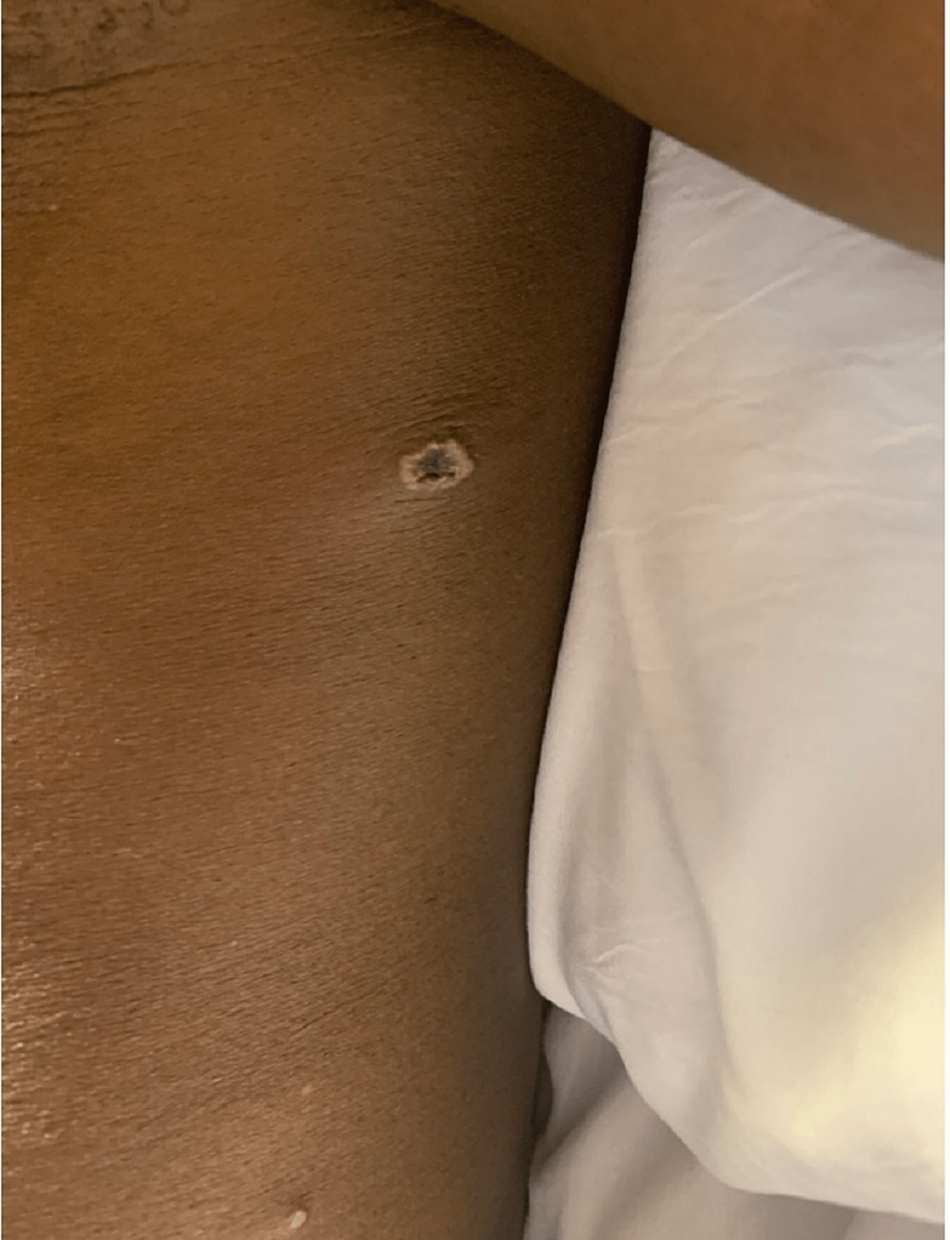
MPVX rash: ulcerated lesion, 4 mm in diameter MPVX: monkeypox virus, mm: millimeter

**Figure 4:**
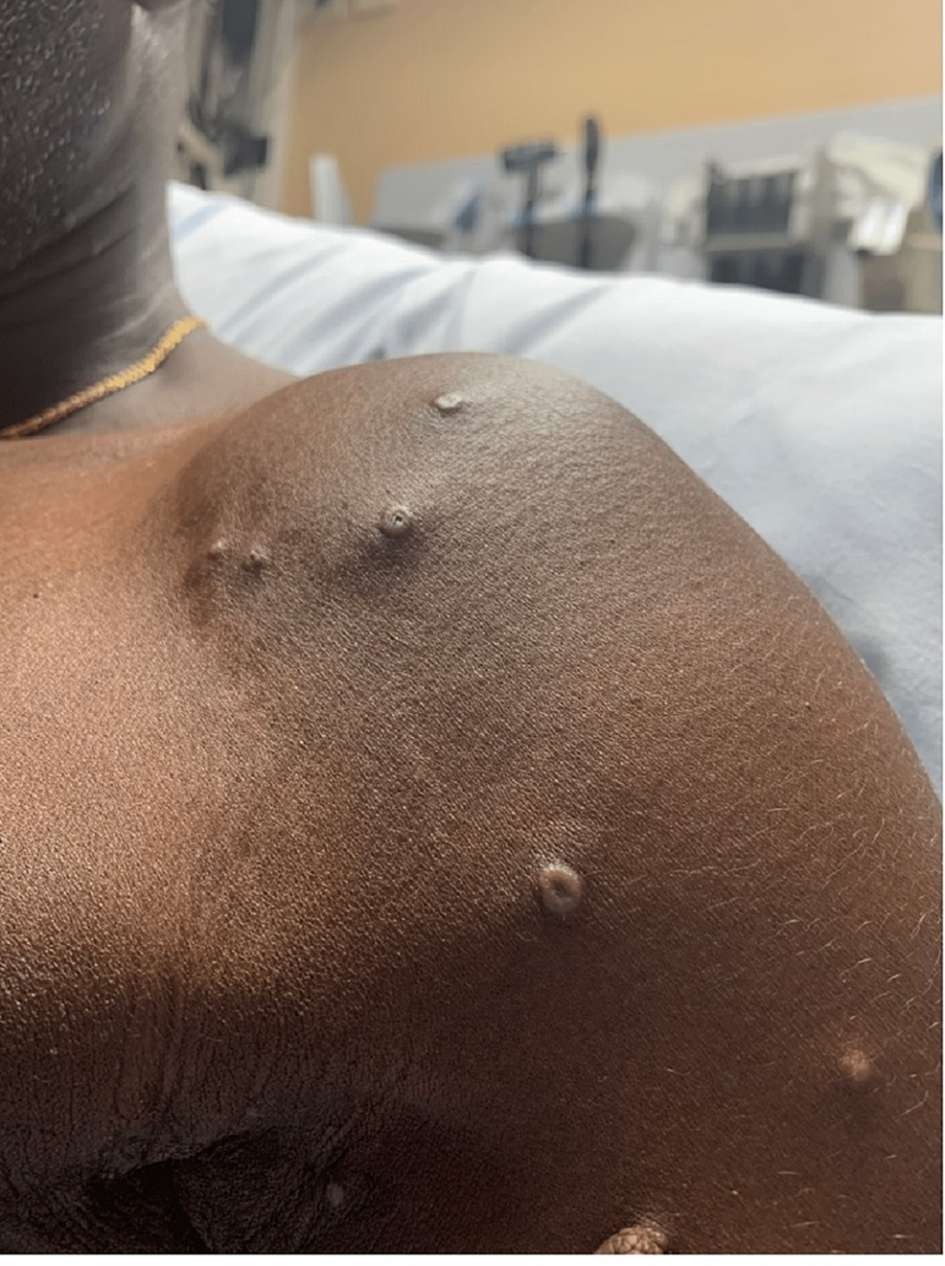
MPVX rash: multiple umbilicated pustules, 3-4 mm in diameter MPVX: monkeypox virus, mm: millimeter

A 51-year-old African male presented with a rash (Figures 1-4). The patient was in Nigeria for three weeks before returning to the United States. He came straight to the emergency room of a community hospital from the airport after noticing an extensive rash all over his body, which developed during the flight (Figures 1-4). He admitted to ignoring the rash that developed from his hands over the last four days. The patient started feeling feverish one week prior to the development of the rash. The rash was not painful or itchy, except for the lesion on the right penile base. The patient did not have any cough, shortness of breath, chest pain, pain in the abdomen, nausea, vomiting, diarrhea, constipation, headache, blurry vision, or weakness in any extremity. There was also no known contact with anyone presenting with a similar rash, and he was in a monogamous relationship with his wife. He also denied any history of male-to-male intercourse. Various lesions over the arms, chest, back, lower extremities, and genitals were present upon thorough dermatological examination. Some lesions looked like pustules, and some were little, larger with scrapped centers. The expert opinion suggested serological testing for chicken pox and MPXV as the lesions were in different stages (Table 1). The patient was asked to quarantine until all lesions have dried up. Application of 2% topical mupirocin ointment thrice a day was prescribed for the lesions, and 25 milligrams of hydroxyzine tablets were given thrice daily.

**Table 1:** Serological test results of the patient RT-PCR: reverse transcriptase-polymerase chain reaction, DNA: deoxyribonucleic acid

Tests	Results	
Varicella-Zoster antibody	1,701.00 index	Positive
Varicella-Zoster IgM assay	<0.90	Negative
Referred test	Lesion specimen	RT-PCR positive for the detection of West African monkeypox
Varicella-Zoster virus DNA PCR	Not detected	

At the two-week follow-up, the patient was feeling better, and all his skin lesions have completely healed. There were no open lesions or scabs. He was asked to follow up if he noticed any similar lesions or a general worsening of his overall condition, which never happened.

## Discussion

As our patient recently traveled from Nigeria, it is to be noted that MPXV originated from different parts of Central and West Africa [4]. It is primarily spread when a person comes into touch with infected animals or people who have lesions from the virus on their skin [5]. Recently, in 2022, between May 7 and 25, a total of 86 MPXV cases were reported in the United Kingdom, of which 66 cases involved gay or bisexual or other men who have sex with men [6]. However, few reports of respiratory transmission have also been seen in some parts of West Africa [7]. MPXV infection presents with prodromal features of fever and lymphadenopathy, followed by a rash on the face and other regions of the body. The dermatological presentation has five stages: macular stage, papule phase, vesicular phase, pustular phase, and scab phase [8,9]. Monkeypox rashes are mostly painful and itchy and are considered contagious until all the lesions are crusted and new skin has formed. Clinical manifestation of an MPXV infection can vary, with some presenting as rash as the only clinical manifestation. The number of skin lesions and lymph nodes, involvement of anatomical areas resulting in serious sequelae that include scarring or strictures, and involvement of multiple organ systems are some of the factors depicting the severity of the disease.

Basgoz et al. described some of the common skin conditions that can have a variable dermatological clinical presentation like MPXV [10]. It included genital herpes, varicella infection, human immunodeficiency virus (HIV), molluscum contagiosum, gonorrhea, syphilis, lymphogranuloma venereum (LGV), and chancroid [10]. Herpes zoster or shingles rash is painful and pruritic in nature, whereas rash in the primary and secondary stages of syphilis is non-painful and non-pruritic. Boesecke et al. reported an unusual case of severe MPXV infection harboring uncontrolled HIV and syphilis infection [11]. It was after the treatment with 600 mg oral tecovirimat for seven days along with antiretroviral therapy (ART) regimen bictegravir, emtricitabine, and tenofovir alafenamide (TAF) and 2 g intravenous ceftriaxone for syphilis for 10 days that his MPXV lesions resolved [11]. The British HIV Association (BHIVA) also has suggested that HIV patients with clusters of differentiation (CD4) count below 200 cells per cubic millimeter and persistent detectable viral load of more than 200 copies per milliliter or a recent HIV-related illness should be considered at higher risk for MPXV [12].

Polymerase chain reaction (PCR) is the definitive diagnostic method for MPXV [12], and its management is mainly supportive treatment in the majority of cases. The severe form of infection with extensive rash or any associated sepsis is warranted with antiviral treatment such as tecovirimat [13]. Currently, two live vaccinia viruses, the smallpox vaccine ACAM2000 and the smallpox/monkeypox vaccine JYNNEOS, are used for pre-exposure and post-exposure prophylaxis against monkeypox [14]. In 2019, the Food and Drug Administration (FDA) licensed JYNNEOS for the prevention of smallpox and monkeypox disease in adults aged ≥18 years determined to be at high risk for infection with these viruses. JYNNEOS is administered subcutaneously as a two-dose series delivered 28 days apart [14]. Myopericarditis has been reported with the use of ACAM2000 [14]. Suspected and confirmed cases must be quarantined until the complete resolution of skin lesions. The rise in the incidence of this zoonotic infection needs further evaluation and consideration with additional diagnostic molecular studies to better understand the mechanism involved in disease transmission and spread.

## Conclusions

Recently, a steep rise in MPXV individuals globally has been reported by the WHO. Varied clinical presentation and its prevalence in the uncontrolled HIV population warrant further research. Basic precautionary measures such as avoiding skin-to-skin contact with persons having a rash similar to monkeypox and maintaining good hand hygiene can have a huge impact in preventing this rare disease from turning into a pandemic.
